# The survivin/XIAP suppressant YM155 impairs clonal growth and induces apoptosis in JAK2^V617F^ cells

**DOI:** 10.1016/j.htct.2024.05.012

**Published:** 2024-09-07

**Authors:** Jorge Antonio Elias Godoy Carlos, Keli Lima, Eduardo Magalhães Rego, Leticia Veras Costa-Lotufo, João Agostinho Machado-Neto

**Affiliations:** a Department of Pharmacology, Institute of Biomedical Sciences, University of São Paulo, São Paulo, Brazil; b Laboratory of Medical Investigation in Pathogenesis and Targeted Therapy in Onco-Immuno-Hematology (LIM-31), Department of Internal Medicine, Hematology Division, Medical School, University of São Paulo, São Paulo, Brazil

**Keywords:** Myeloproliferative neoplasms, Inhibitor of apoptosis proteins, Surviving, XIAP, YM155

## Abstract

The central role of the control of apoptosis in the pathophysiology of Philadelphia chromosome-negative myeloproliferative neoplasms has recently been reinforced in genetic and pharmacological studies. The inhibitor of apoptosis protein family has eight members and plays an important role in apoptosis, with the most studied being survivin (BIRC5) and X-linked inhibitor of apoptosis (XIAP). YM155 is a small molecule with antineoplastic potential that has been described as a suppressant of survivin and XIAP. In the present study, *BIRC5* expression was significantly increased in primary myelofibrosis patients compared to healthy donors. On the other hand, *XIAP* expression was reduced in myeloproliferative neoplasms patients. In JAK2^V617F^ cells, YM155 reduces cell viability and autonomous clonal growth and induces apoptosis, cell cycle arrest, and autophagy. HEL cells that show greater malignancy are more sensitive to the drug than SET2 cells. In the molecular scenario, YM155 modulates apoptosis-, cell cycle-, DNA damage- and autophagy-related genes. Protein expression analysis corroborates the observed cellular phenotype and exploratory gene expression findings. In summary, our results indicate that survivin/BIRC5 and XIAP are differently expressed in myeloproliferative neoplasms and YM155 has multiple antineoplastic effects on JAK2^V617F^ cells suggesting that inhibitor of apoptosis proteins may be a target for pharmacological interventions in the treatment of these diseases.

## Introduction

Philadelphia chromosome-negative myeloproliferative neoplasms (MPNs) comprise a group of diseases the main characteristics of which are exacerbated proliferation, resistance to cell death, and maintenance of terminal differentiation of hematopoietic progenitors.^1^ The most common MPNs include essential thrombocythemia (ET), polycythemia vera (PV), and primary myelofibrosis (PMF).^1^^,^^2^ During the course of the disease, MPN patients may progress to bone marrow failure or acute myeloid leukemia (AML) transformation, which presents a dismal diagnosis.^1^^,^^3^ From a molecular point of view, MPNs present a high incidence of JAK2 activating mutations (i.e., JAK2^V617F^, *indel* CALR, and MPL mutations), which result in constitutive activation of the JAK2/STAT signaling pathway and represents an important molecular target for disease treatment and control.^2^^,^^4^ Ruxolitinib, a selective JAK1/2 inhibitor, is approved for the treatment of PMF and PV patients however despite providing clinical benefits, it does not lead to a complete remission and fails to eliminate malignant clones.^5^, ^6^, ^7^, ^8^

Recently, a central role of the control of apoptosis in the phenotype of MPNs has been demonstrated: inhibition of multiple members of the BCL2 family was able to recapitulate effects similar to those seen with JAK2 inhibitors.^9^ In fact, the BCL2 family proteins, in particular BCL2 and BCL2L1 (also known as BCL-XL) are transcriptional targets of STAT3/5.^10^, ^11^, ^12^ In this sense, another family of proteins that play an important role in the control of apoptosis is the inhibitor of apoptosis protein (IAP) nevertheless its role is little explored in the context of MPNs. The IAP family has eight members, the most studied being survivin (BIRC5) and X-linked inhibitor of apoptosis (XIAP).^13^ The canonical function of IAP is to bind and inhibit activated caspases and prevent apoptosis, which is mainly performed by the baculovirus IAP Repeat (BIR) domain. However, due to other protein domains that these proteins carry, other functions and/or cellular locations can be attributed depending on the cellular context and molecular background.^13^, ^14^, ^15^

Given the relevance of IAPs in cancer, multiple pharmacological inhibitors have been proposed with promising preclinical results.^16^, ^17^, ^18^ YM155 (sepantronium bromide) is a small molecule with antineoplastic potential that has been described as a suppressant of survivin and XIAP . Herein, we describe the expression of survivin/BIRC5 and XIAP in ET, PV, and PMF patients and JAK2^V617F^ cell lines and the molecular and cellular effects of treatment with YM155 in MPN cellular models.

## Material and methods

### Gene expression data

*BIRC5* (median of probes 202,094_at and 202,095_s_at) and *XIAP* (median of probes 206,536_s_at, 206,537_at, 225,858_s_at, 225,859_at, 235,222_x_at, and 235,222_x_at) mRNA expression data from blood samples of healthy donors (*n* = 21), and ET (*n* = 19), PV (*n* = 41), and PMF patients (*n* = 9) were obtained from GEOR2 (https://www.ncbi.nlm.nih.gov/geo/geo2r/; GEO accession GSE26049).

### Cell culture and inhibitors

HEL cells were kindly provided by Prof. Dr. Sara Teresinha Olalla Saad (Hemocentro, University of Campinas, Campinas, Brazil). Additionally, SET2 cells were kindly provided by Prof. Dr. Fabíola Attié de Castro (School of Pharmaceutical Sciences of Ribeirão Preto, University of São Paulo, Ribeirão Preto, Brazil). SET2 and HEL cells harboring the JAK2^V617F^ mutation were tested and authenticated by short tandem repeat (STR) matching analysis using the PowerPlex® 16 HS system (Promega, Madison, WI, USA) and the ABI 3500 Sequence Detector System (Life Technologies, Foster City, CA, USA). Cell culture conditions were created following the recommendations of the American Type Culture Collection (ATCC) and Deutsche Sammlung von Mikroorganismen und Zellkulturen (DSMZ). All cell lines were mycoplasma free. Primary CD34^+^ cells were obtained from the peripheral blood of a healthy donor using MIDI-MACS immunoaffinity columns (Miltenyi Biotec, Auburn, CA, USA). Samples were obtained after obtaining informed consent and the study protocol was approved by the Ethics Committee of the Institute of Biomedical Sciences of the University of São Paulo (CAAE:39,510,920.1.0000.5467) following the Declaration of Helsinki. YM155 was obtained from Cayman Chemical (Ann Arbor, MI, USA) and ruxolitinib was obtained from InvivoGen (San Diego, CA, USA).

### Western blot analysis

Total protein extraction was achieved using a buffer containing 100 mM Tris (pH 7.6), 1 % Triton X-100, 2 mM phenylmethylsulfonyl fluoride (PMSF), 10 mM Na_3_VO_4_, 100 mM NaF, 10 mM Na_4_P_2_O_7_, and 4 mM ethylenediaminetetraacetic acid (EDTA). Equal amounts of protein (30 μg) from the samples were subjected to sodium dodecyl-sulfate polyacrylamide gel electrophoresis (SDS-PAGE) in an electrophoresis device, followed by electrotransfer of the proteins to nitrocellulose membranes. The membranes were blocked with 5 % non-fat dry milk and incubated with specific primary antibodies diluted in blocking buffer, followed by secondary antibodies conjugated to horseradish peroxidase (HRP). Western blot analysis was performed using a SuperSignalTM West Dura extended Duration Substrate system (Thermo Fisher Scientific) and a G:BOX Chemi XX6 gel document system (Syngene). Antibodies against survivin/BIRC5 (#2808S), phospho(p)-ribosomal protein S6 (RPS6; #4858), RPS6 (#2217), p-ULK1 (#14,202), ULK1 (#8054), SQSTM1/p62 (#88,588), LC3B (#2775), PARP1 (#9542), γH2AX (#9718), actin (#8456), and α-tubulin (#2144) were obtained from Cell Signaling Technology (Danvers, MA, USA). Antibodies against XIAP (ab28151) were obtained from Abcam (Cambridge, MA, USA). Band intensities were determined using UN-SCAN-IT gel 6.1 software (Silk Scientific; Orem, UT, USA).

### Cell viability assay

Cell viability was measured using the methylthiazoletetrazolium (MTT) assay. HEL (2  ×  10^4^ cells/well) and SET2 cells (4  ×  10^4^ cells/well) were cultured in a 96-well plates in Roswell Park Memorial Institute (RPMI) medium containing 10 % or 20 % fetal bovine serum (FBS), respectively, in the presence of a vehicle or graded concentrations of YM155 (0.0032, 0.016, 0.08, 0.4, 2, 10, and 50 μM) for 24, 48, and 72 hours. Half-maximal inhibitory concentration (IC_50_) values were calculated using the nonlinear regression analysis of GraphPad Prism 5 (GraphPad Software, Inc., San Diego, CA, USA). For combined treatment analysis, HEL and SET2 cells were exposed to graded concentrations of YM155 (0.08, 0.16, 0.32, 0.64, 1.25, 2.5, and/or 5  μM) and ruxolitinib (3, 10, 30,100, 300, and 1000 nM) alone or in combination for 48 hours. Data are illustrated using multiple experiment viewer (MeV) 4.9.0 software (http://www.tm4.org/mev/).

### Autonomous colony formation assay

Colony formation assays were performed in semi-solid methylcellulose medium (1  ×  10^3^ cells/mL in MethoCult 4230: StemCell Technologies Inc., Vancouver, BC, Canada) in the presence of a vehicle or YM155 (0.04, 0.08, 0.15, 0.3, and 0.6 μM). After eight days (HEL cells) or twelve days (SET2 cells) of culture, colonies were detected by adding a MTT solution (5 mg/mL). Images were acquired using the G:BOX Chemi XRQ (Syngene, Cambridge, UK) and analyzed using ImageJ software (US National Institutes of Health, Bethesda, MD, USA).

### Quantitative reverse transcription polymerase chain reaction (qRT-PCR)

Total RNA was extracted using the TRIzol reagent (Thermo Fisher Scientific) and DNA was synthesized from 1 μg RNA using a High-Capacity cDNA Reverse Transcription Kit (Thermo Fisher Scientific). Quantitative PCR (qPCR) was performed using a QuantStudio 3 Real-Time PCR System in conjunction with a SybrGreen System (Thermo Fisher Scientific). *HPRT1* and *ACTB* were used as reference genes. Relative quantification values were calculated using the 2^-ΔΔCT^ equation.^19^ Expression of cell cycle-, apoptosis-, DNA-damage-, and autophagy-related genes (Supplementary Table 1) were investigated in HEL and SET2 cells upon a vehicle or YM155 (2.5 and 5 μM, respectively) exposure for 24 h. A Heatmap was drawn using the multiple experiment viewer (MeV) 4.9.0 software (http://mev.tm4.org). Differentially expressed genes that presented *p*-values <0.05 were used for network construction by the GeneMANIA database (https://genemania.org/).

### Cell death assay

In total, HEL and SET2 cells (1 × 10^5^ per well) were seeded in 24-well plates in the presence of a vehicle or YM155 (0.32, 0.64, 1.25, 2.5, 5, and 10 μM) for 48 h. Next, the cells were washed with ice-cold phosphate-buffered saline (PBS) and resuspended in a binding buffer containing 1 μg/mL propidium iodide (PI) and 1 μg/mL APC-labeled annexin V. All specimens were analyzed by flow cytometry (FACSCalibur; Becton Dickinson, Franklin Lakes, New Jersey, USA) after incubation for 15 min at room temperature in a light-protected area. Ten thousand events were recorded for each sample.

### Cell cycle assay

In total, HEL and SET2 cells (1 × 10^5^ per well) were seeded in 12-well plates in the presence of a vehicle or YM155 (0.32, 0.64, 1.25, 2.5, 5, and 10 μM), harvested at 48 h, fixed with 70 % ethanol, and stored at 4 °C for at least 4 h. Next, the fixed cells were stained with PBS containing 20 μg/mL propidium iodide (PI) and 10 μg/mL RNase A for 30 min at room temperature in a light-protected area. The DNA content distribution was determined using flow cytometry (FACSCalibur; Becton Dickinson, FranklinLakes, NJ, USA) and analyzed using FlowJo software (Treestar, Inc. San Carlos, CA, USA).

### Statistical analysis

Statistical analyses were performed using GraphPad Prism 8 (GraphPad Software Inc.). The Kruskal-Wallis test and Dunn post-hoc or ANOVA and Bonferroni post-test, or Student *t*-test, were used, as appropriate. All *p*-values were two-sided, and the significance level was set to 5 %.

## Results

### BIRC5 and XIAP mRNA levels are differently expressed in myeloproliferative neoplasms

**Figure 1:**
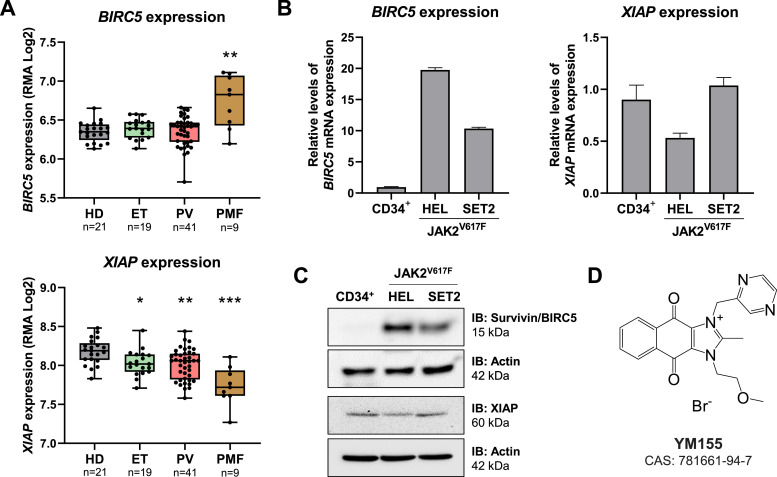
Survivin and XIAP are differently expressed in myeloproliferative neoplasm patients and cellular models. (A) Gene expression data was obtained from GEOR2 (https://www.ncbi.nlm.nih.gov/geo/geo2r/; GEO accession GSE26049) for blood samples from polycythemia vera (PV), essential thrombocythemia (ET), and primary myelofibrosis (PMF) patients. The median of *BIRC5* (202,094_at and 202,095_s_at) and *XIAP* (206,536_s_at, 206,537_at, 225,858_s_at, 225,859_at, 235,222_x_at, and 235,222_x_at) data obtained for each patient was used and number of patients are indicated; *p-value *<*0.05, **p-value *<*0.01, ***p-value *<*0.001; Kruskal-Wallis test and Dunn's post-test. (B) qPCR analysis of *BIRC5* and *XIAP* mRNA expression in normal CD34^+^, HEL, and SET2 cells. (C) Western blot analysis for survivin and XIAP in total cell extracts from normal CD34^+^, HEL, and SET2 cells; membranes were reprobed with the antibody for the detection of actin, and developed with the SuperSignal™ West Dura Extended Duration Substrate system using a G:BOX Chemi XX6 gel document system. (D) Chemical structure of the pharmacological survivin/XIAP suppressant YM155.

Taking advantage of publicly available gene expression data, the expression of *BIRC5* and *XIAP* were investigated in samples from healthy donors and from patients with ET, PV, and PMF. *BIRC5* expression was significantly higher in PMF patients compared to healthy donors (*p*-value <0.05, Figure 1A). On the other hand, *XIAP* expression was reduced in MPN patients (all *p*-values <0.05, Figure 1A). Next, the expression of BIRC5 and XIAP were investigated in cellular models with JAK2^V617F^ mutations. BIRC5 expression was higher in HEL and SET2 cell lines when compared to normal hematopoietic cells (10- to 20-fold), whereas XIAP expression was similar between tested samples, being slightly lower in HEL cells (Figure 1B-C). The JAK2^V617F^ cell models used differ in respect to the disease of origin, proliferation rate, and sensitivity to JAK2 inhibitors. HEL cells are more malignant, being derived from erythroleukemia, have rapid proliferation and intrinsic resistance to JAK2 inhibitors^20^, while SET2 cells are derived from an AML secondary to ET, which have a lower proliferation rate and sensitivity to JAK2 inhibitors.^21^^,^^22^ To test whether this differential expression of BIRC5 and XIAP could be a vulnerability in MPN models, YM155, a pharmacological suppressant of survivin and XIAP (Figure 1D), was used in JAK2^V617F^-mutated cell lines.

### YM155 reduces cell viability and autonomous clonal growth in HEL and SET2 cells

**Figure 2:**
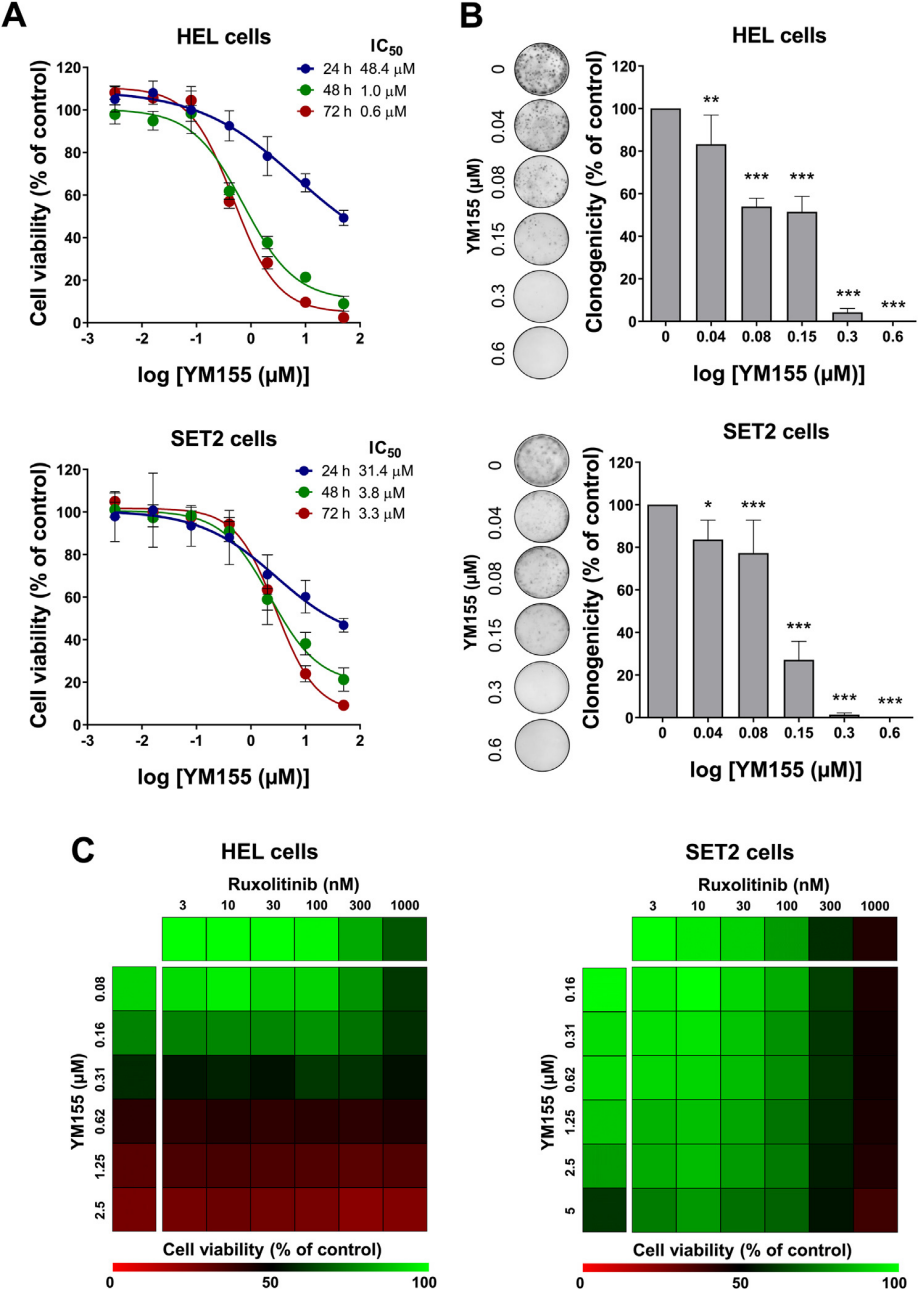
YM155 reduces cell viability and autonomous clonal growth in JAK2^V617F^ cellular models. (A) Dose- and time-response cytotoxicity was analyzed by methylthiazoletetrazolium (MTT) assay for HEL and SET2 cells treated with graded concentrations of YM155 (ranging from 0.0032 to 50 µM) for 24, 48, and 72 h. Values are expressed as the percentage of viable cells for each condition relative to vehicle-treated controls. Results are shown as the mean ± standard deviation (SD) of at least three independent experiments. (B) Colonies containing viable cells were detected by adding an MTT reagent after 8–12 days of culturing the cells in presence of vehicle or YM155 (0.04, 0.08, 0.16, 0.3, and 0.6 µM). Colony images are shown for one experiment and bar graphs show the mean ± SD of at least three independent experiments. *p-value <0.05, **p-value <0.01, ***p-value <0.001; ANOVA test and Bonferroni post-test*.* (C) Dose-response cytotoxicity for combined treatment was analyzed by methylthiazoletetrazolium (MTT) assay for JAK2^V617F^ cells treated with graded concentrations of YM155 (HEL: ranged from 0.08 to 2.5 μM, SET2: ranged from 0.16 to 5 μM) and ruxolitinib (ranged from 3 to 1000 nM) alone or in combination for 48 hours, as indicated. Values are expressed as the percentage of viable cells for each condition relative to vehicle-treated controls. Results are shown as the mean of at least three independent experiments.

First, the concentration- and time-dependent effects of YM155 on cell viability in JAK2^V617F^ cells were determined. HEL cells showed greater sensitivity to YM155 when compared to SET2 cells. IC_50_ values for HEL cells were 48.4, 1.0, and 0.6 µM, and for SET2 cells they were 31.4, 3.8, and 3.3 µM for 24, 48, and 72 h, respectively (Figure 2A). Of note, long-term exposure to YM155 strongly reduced the capacity for autonomous clonal growth, with a > 90 % reduction at the 0.3 µM concentration in both cell lines evaluated (*p*-value *<*0.05, Figure 2B). The effects of combined treatment with YM155 and ruxolitinib on HEL and SET2 cells were also evaluated. In both cell lines, synergic, additive, or antagonistic effects were not observed in the combination of drugs, indicating that the drugs can be used together without gains or losses in individual effects (Figure 2C).

### Apoptosis-, cell cycle-, DNA damage- and autophagy-related genes are modulated by YM155 in JAK2^V617F^ cells

**Figure 3:**
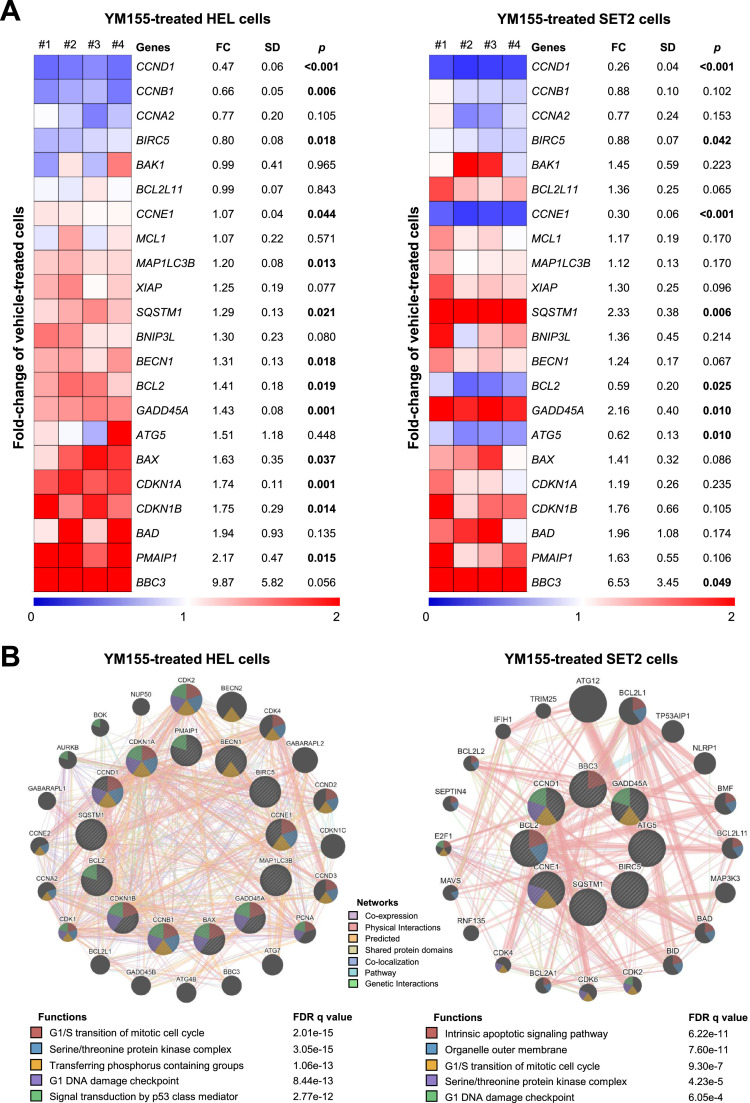
YM155 modulates apoptosis-, cell cycle-, DNA damage- and autophagy-related genes in HEL and SET2 cells. (A) Heatmap depicting the gene expression profile of JAK2^V617F^ cells treated with a vehicle or YM155 (HEL: 2.5 μM, SET2: 5 μM) for 24 h. The blue color on the heatmap indicates decreased mRNA levels while red indicates induced mRNA levels, which were normalized by vehicle-treated cells (*n* = 4). The fold-change (FC) to vehicle-treated cells, standard deviation (SD) and *p*-values are described, Student *t*-test. (B) Network for YM155 modulated genes constructed using the GeneMANIA database (https://genemania.org/) for HEL and SET2 cells. Genes significantly modulated are illustrated as crosshatched circles; the interacting genes included by modeling the software are indicated by circles without crosshatched. The main biological interactions, associated functions, and false discovery rate (FDR) q values are described in the Figure.

Next, a panel of genes related to apoptosis, cell cycle, DNA damage, and autophagy were investigated to understand the mechanisms involved in YM155-induced cell viability reduction in JAK2^V617F^ cells. In HEL cells, 13 of out 22 investigated genes were modulated (*BCL2, BAX, PMAIP1, CCND1, CCNE1, CCNB1, CDKN1A, CDKN1B, GADD45A, MAP1LC3B, SQSTM1, BECN1*, and *BIRC5;* all *p*-values <0.05; Figure 3A), whereas in SET2 cells 8 of out 22 investigated genes were modulated (*CCND1, BIRC5, CCNE1, SQSTM1, BCL2, ATG5, GADD45A*, and *BBC3*; all *p*-values *<*0.05; Figure 3A). Among the cellular and molecular processes associated with genes modulated by YM155 in HEL and SET2 cells, the most noteworthy are G1/S transition of mitotic cell cycle, G1 DNA damage checkpoint, intrinsic apoptotic signaling pathway, outer organelle membrane, serine/threonine protein kinase complex, transferring phosphorus-containing groups, signal transduction by p53 class mediator, and serine/threonine protein kinase complex (all false discovery rates (FDRs) <0.05; Figure 3B).

### YM155 induces apoptosis and cell cycle delay in JAK2^V617F^-mutated cells

**Figure 4:**
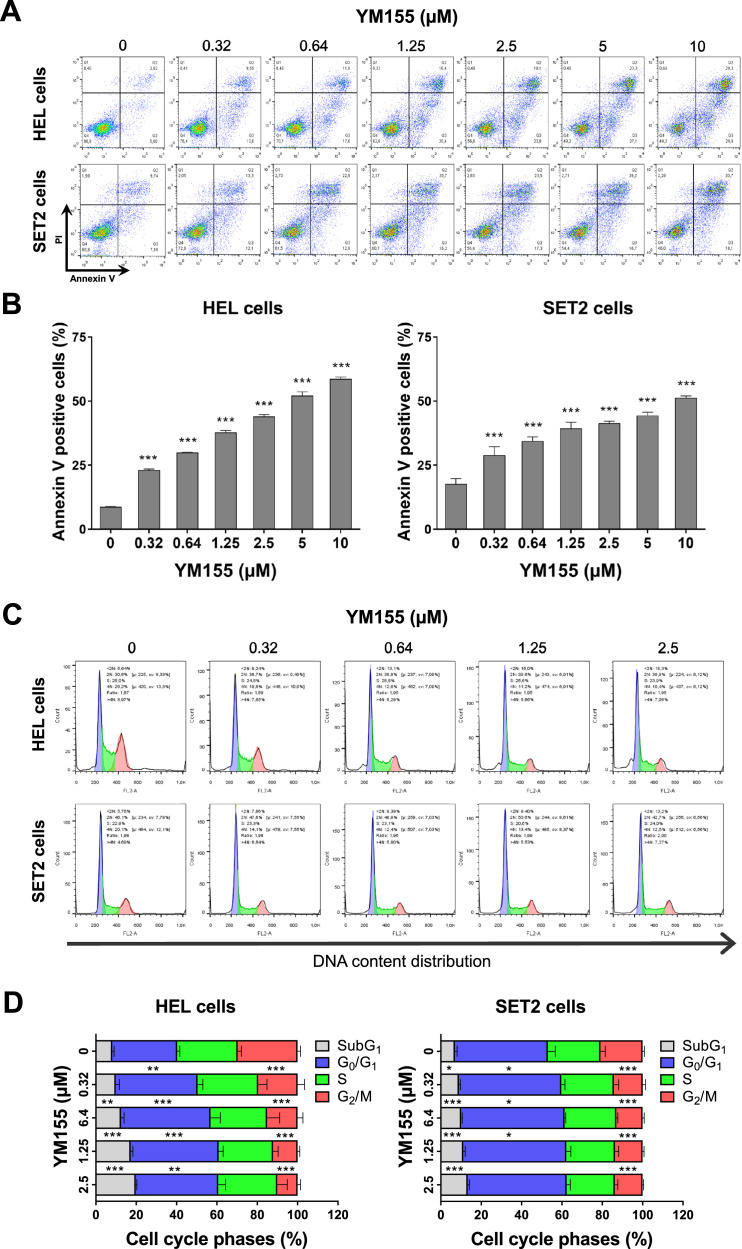
YM155 induces cell death and cell cycle delay in HEL and SET2 cells. (A) Cell death was detected by flow cytometry in JAK2^V617F^ cells treated with vehicle or with increasing concentrations of YM155 (0.32, 0.64, 1.25, 2.5, 5, and 10 μM) for 48 h using an APC-annexin V/ propidium iodide (PI) staining method. Representative dot plots are shown for each condition. The upper and lower right quadrants (Q2 plus Q3) cumulatively contain the cell death population (annexin *V*^+^ cells). Bar graphs represent the mean ± standard deviation (SD) of at least three independent experiments. The *p*-values and cell lines are indicated in the graphs; *** *p*-value <0.0001; ANOVA and Bonferroni post-test. (B) Phases of the cell cycle were determined by analyzing the DNA content by staining with propidium iodide and acquiring the data by flow cytometry following exposure of JAK2^V617F^ cells to the vehicle or YM155 (0.32, 0.64, 1.25, and 2.5 μM) for 48 h. A representative histogram for each condition is illustrated. The mean ± SD of at least three independent experiments is represented in the bar graph. The *p*-values and cell lines are indicated in the graphs; * *p*-value <0.05; ** *p*-value <0.01; *** *p*-value <0.001, ANOVA and Bonferroni post-test.

Given the results obtained in the gene expression analysis, the effects of YM155 on the induction of apoptosis and cell cycle progression were investigated. In HEL and SET2 cells, YM155 induced apoptosis in a dose-dependent manner (*p*-value <0.05, Figure 4A). At lower concentrations, YM155 induced a cell cycle delay as seen by the increase in the percentage of cells in the G_0_/G_1_ phases (*p*-value <0.05). At higher concentrations, the drug-induced cell death and/or DNA damage as evidenced by the increase in cells in subG_1_ phase (*p*-value <0.05, Figure 4B). Consistent with the cell viability results, these phenotypic cellular events were more pronounced in HEL cells than in SET2 cells.

### YM155 triggers autophagy, apoptosis, and DNA damage markers in HEL and SET2 cells

**Figure 5:**
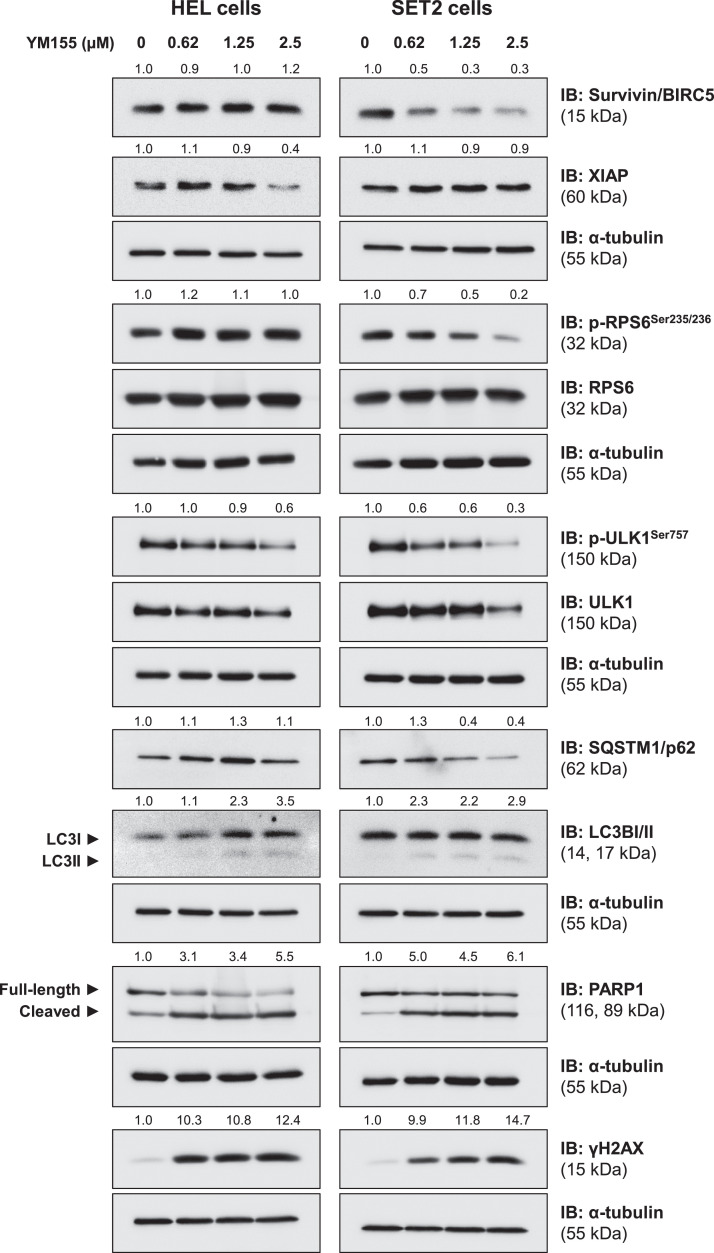
YM155 induces autophagy, apoptosis, and DNA damage markers in JAK2^V617F^ cells. Western blot analysis for survivin/BIRC5, XIAP, phospho(p)-ribosomal protein S6 (RPS6), RPS6, p-ULK1, ULK1, SQSTM1/p62, LC3BI/II, PARP1 (total and cleaved), and γH2AX in total extracts from HEL and SET2 cells treated with a vehicle or with increasing doses of YM155 (0.62, 1.25, and 2.5 µM) for 24 h. Membranes were re-incubated with α-tubulin antibody and developed with the SuperSignal™ West Dura Extended Duration Substrate system and GBox. Relative quantification was performed using band intensities determined by UN-SCAN-IT gel 6.1 software. The expression of XIAP, survivin/BIRC5, SQSTM1/p62, and γH2AX was normalized by α-tubulin. Ratios for p-RPS6/RPS6, p-ULK1/ULK1, LC3BII/LCBI, and cleaved PARP1/full-length PARP1 are also described.

Finally, the effects of YM155 on drug targets (survivin/BIRC5 and XIAP) and key molecular markers of related pathways (RPS6), autophagy (ULK1, SQSTM1/p62, and LC3B), apoptosis (cleaved-PARP1) and DNA damage (γH2AX) were evaluated. In HEL, but not in SET2 cells, YM155 reduced XIAP expression. On the other hand, survivin and p-RPS6 were reduced in SET2, but not in HEL cells. In both JAK2^V617F^ cells, YM155 induced autophagy markers, including reductions of phosho-ULK1 and SQSTM1/p62, and increases of LC3BII, which was more prominent in SET2 cells. Apoptosis and DNA damage markers were strongly induced in both cell lines (Figure 5). Together these molecular findings corroborate our exploratory data on gene expression and the observed cellular events, in addition to providing insights for the interpretation of differences in observed YM155 potency between the cellular models used.

## Discussion

Herein, we have reported the expression of survivin/BIRC5 and XIAP in MPN patients and the underlying cellular and molecular antineoplastic effects of YM155 on MPN cellular models. In this study, survivin/BIRC5 expression was increased in patients with PMF, a group of MPN patients with poor prognoses. On the other hand, XIAP expression was reduced in MPN patients. In a previous study, increased survivin/BIRC5 expression was reported in megakaryocytes from MPN patients (ET, PV, and PMF) compared to healthy donors, which was independent of the presence of JAK2^V617F^ and CALR *indel* mutations.^23^ Similarly, in patients with chronic myelomonocytic leukemia, one group of MPN, survivin/BIRC5 expression was increased; this was associated with a higher evolution rate and shorter survival.^24^ Regarding XIAP expression, the findings of the present study agree with a previous study that demonstrated that hematopoietic cells derived from JAK2^V617F^-mutated mice have lower levels of XIAP compared to cells from wild-type JAK2 mice, as was also observed in samples from patients with MPNs compared to normal CD34^+^ cells.^25^ The reduction of XIAP was associated with the repropagation of the TNF response caused by the JAK2^V617F^ mutation via TNFR2.^25^

YM155 is a small imidazolium-based compound that has been reported to block the gene expression of survivin/BIRC5 via its promoter inhibition and inhibits XIAP by degradation induction.^26^, ^27^, ^28^ However, the effects of YM155 seem to differ in different cancer models and cellular backgrounds, for example, in some studies it has been shown that YM155 may directly induce DNA damage and that the reduced expression of survivin and XIAP may rather be a consequence of DNA damage due to the interrelationship between these IAP and DNA damage repair pathways.^26^^,^^29^^,^^30^ Thus, defining the effects of YM155 in models with the oncogenic molecular background caused by the JAK2^V617F^ mutation (constitutive activation of the JAK2/STAT pathway) may contribute to the potential repositioning of the drug in this context. In this study, both effects of YM155 were observed: a reduction of XIAP protein levels without alteration in gene expression (e.g*.* induction of degradation) in HEL cells, and a modest reduction (around 12–20 %; *p*-value *<*0.05) of *BIRC5* gene expression in JAK2^V617F^ evaluated cell lines, but with a prominent decrease in protein levels only in SET2 cells. These findings illustrate the complexity of YM155 action in different cell types, even with similar molecular backgrounds. The HEL cell line, originating from a patient with erythroleukemia, exhibits accelerated growth in culture and displays reduced sensitivity to ruxolitinib, even when administered at doses capable of inhibiting JAK2/STAT activation.^20^ Conversely, the SET2 cell line, derived from a patient experiencing leukemic transformation of ET, demonstrates slower proliferation, heightened responsiveness to ruxolitinib, and a dependency on elevated FBS concentrations for optimal growth.^21^ These inherent distinctions between HEL and SET2 cell lines could potentially influence their respective responses to YM155. Of note, in both JAK2^V617F^ cellular models, YM155 led to increases in DNA damage markers, as well as the expression of cell cycle checkpoint-related genes.

Our molecular analyzes also indicated strong reductions of cyclin D (*CCND1*) and cyclin E (*CCNE1*) upon exposure to YM155 in JAK2^V617F^ cells. Cyclin D is the first cyclin to be induced during cell cycle entry, followed by cyclin E induction ^31^. The CDK4/6-cyclin D and CDK2-cyclin E complexes lead to retinoblastoma protein phosphorylation which is the first checkpoint to signal cell cycle entry.^31^ In the context of MPN, it is known that the constitutive activation of the JAK2/STAT signaling pathway leads to the expression of these cyclins and promotes exacerbated proliferation.^32^ Genes expressed during cell cycle checkpoints and in response to DNA damage such as p21 (*CDKN1A*), p27 (*CDKN1B*), and/or *GADD45A* were induced and may contribute to the arrest of cell cycle progression.^33^^,^^34^ Indeed, a cell cycle delay was confirmed in JAK2^V617F^ cells after exposure to the drug, indicating that at lower doses a cytostatic effect is observed.

Autophagy, another YM155-associated cell death mechanism,^27^^,^^35^ was modestly induced in HEL and SET2 cells, as evidenced by reduced ULK1 phosphorylation, consumption of SQSTM1/p62 and increased LC3BII. Indeed, genes associated with autophagy were induced by drug treatment (i.e. *MAP1LC3B, SQSTM1, BECN1* and *ATG5*). In SET2 cells, a model in which autophagy was more pronounced, a reduction in the activity of the PI3K/AKT/mTOR pathway (decreased RPS6 phosphorylation) was also observed, which may have contributed to this process since mTOR activation acts as a potent inhibitor of autophagy.^36^^,^^37^ On the other hand, apoptosis markers (cleaved PARP1 and phosphatidylserine exposure) and proapoptotic genes induced by DNA damage (i.e. *BAX, PMAIP1* and *BBC3*) were more evident, indicating that this may be the most important mechanism of death associated with loss of cell viability induced by YM155 in JAK2^V617F^ cells.

Finally, a point that drew our attention in the JAK2^V617F^ cells was the strong suppression of autonomous clonal growth (i.e. the absence of growth factors) induced by YM155 at submicromolar concentrations. In patients with AML, autonomous clonal growth was associated with greater disease malignancy and worse clinical outcomes.^38^^,^^39^ The action of YM155 on cancerous clones with greater malignancy may be a point better explored in future studies with MPN models.

YM155 has successfully completed phase I and II clinical trials in solid tumors^40^, ^41^, ^42^, ^43^, ^44^, ^45^, ^46^, ^47^, ^48^ and B-cell lymphoma.^49^^,^^50^ Presently, a study in relapsed/refractory B-cell lymphoma is in the recruitment phase (NCT05263583). Published data from these trials have demonstrated the safety of YM155, albeit with modest or limited efficacy as a single agent. Consequently, there is growing interest in exploring its potential in combination therapies.^41^^,^^42^ In the context of MPNs, this study represents a pioneering effort. Supported by the favorable safety profile of YM155, our preclinical investigations with JAK2^V617F^ models indicate a promising direction for the drug's potential inclusion in forthcoming clinical trials for this disease.

In summary, our results indicate that survivin/BIRC5 and XIAP are differently expressed in MPNs and YM155 has multiple antineoplastic effects on MPN cell models, suggesting that inhibitors of apoptosis proteins may be a point of pharmacological intervention for the treatment of these diseases.

## Conflicts of interest

The authors declare no competing interests.

## Appendix Supplementary materials

Image, application 1
